# Comparison of ^13^C MRI of hyperpolarized [1‐^13^C]pyruvate and lactate with the corresponding mass spectrometry images in a murine lymphoma model

**DOI:** 10.1002/mrm.28652

**Published:** 2021-01-09

**Authors:** Maria Fala, Vencel Somai, Andreas Dannhorn, Gregory Hamm, Katherine Gibson, Dominique‐Laurent Couturier, Richard Hesketh, Alan J. Wright, Zoltan Takats, Josephine Bunch, Simon T. Barry, Richard J. A. Goodwin, Kevin M. Brindle

**Affiliations:** ^1^ Cancer Research UK Cambridge Institute University of Cambridge Cambridge United Kingdom; ^2^ Department of Radiology University of Cambridge, School of Clinical Medicine Cambridge Biomedical Campus United Kingdom; ^3^ Imaging and Data Analytics Clinical Pharmacology and Safety Sciences R&D AstraZeneca Cambridge United Kingdom; ^4^ Department of Digestion, Metabolism and Reproduction Imperial College London Sir Alexander Fleming Building London United Kingdom; ^5^ National Centre of Excellence in Mass Spectrometry Imaging (NiCE‐MSI) National Physical Laboratory Teddington United Kingdom; ^6^ Bioscience, Discovery, Oncology R&D AstraZeneca Cambridge United Kingdom; ^7^ Institute of Infection, Immunity and Inflammation College of Medical, Veterinary and Life Sciences University of Glasgow Glasgow United Kingdom; ^8^ Department of Biochemistry University of Cambridge Cambridge United Kingdom

**Keywords:** hyperpolarized ^13^C MRI, lactate, mass spectrometry imaging, pyruvate, tumor

## Abstract

**Purpose:**

To compare carbon‐13 (^13^C) MRSI of hyperpolarized [1‐^13^C]pyruvate metabolism in a murine tumor model with mass spectrometric (MS) imaging of the corresponding tumor sections in order to cross validate these metabolic imaging techniques and to investigate the effects of pyruvate delivery and tumor lactate concentration on lactate labeling.

**Methods:**

[1‐^13^C]lactate images were obtained from tumor‐bearing mice, following injection of hyperpolarized [1‐^13^C]pyruvate, using a single‐shot 3D ^13^C spectroscopic imaging sequence in vivo and using desorption electrospray ionization MS imaging of the corresponding rapidly frozen tumor sections ex vivo. The images were coregistered, and levels of association were determined by means of Spearman rank correlation and Cohen kappa coefficients as well as linear mixed models. The correlation between [1‐^13^C]pyruvate and [1‐^13^C]lactate in the MRS images and between [^12^C] and [1‐^13^C]lactate in the MS images were determined by means of Pearson correlation coefficients.

**Results:**

[1‐^13^C]lactate images generated by MS imaging were significantly correlated with the corresponding MRS images. The correlation coefficient between [1‐^13^C]lactate and [1‐^13^C]pyruvate in the MRS images was higher than between [1‐^13^C]lactate and [^12^C]lactate in the MS images.

**Conclusion:**

The inhomogeneous distribution of labeled lactate observed in the MRS images was confirmed by MS imaging of the corresponding tumor sections. The images acquired using both techniques show that the rate of ^13^C label exchange between the injected pyruvate and endogenous tumor lactate pool is more correlated with the rate of pyruvate delivery to the tumor cells and is less affected by the endogenous lactate concentration.

## 1 INTRODUCTION

Carbon‐13 (^13^C) MRSI of hyperpolarized ^13^C‐labeled cell substrates enables rapid noninvasive imaging of tissue metabolism in vivo,^1^, ^2^ with lactate dehydrogenase‐catalyzed ^13^C label exchange between injected [1‐^13^C]pyruvate and the endogenous lactate pool being the most studied metabolic reaction. Hyperpolarized [1‐^13^C]pyruvate has been used clinically with promising applications, such as disease staging in prostate cancer,^3^, ^4^ and for detecting tumor treatment response.^5^, ^6^ However, the transient nature of the hyperpolarization means that only relatively rapid metabolic reactions can be studied, and it is difficult to relate signal intensity to concentration, which means that typically only apparent first‐order rate constants describing isotope flux are quoted rather than metabolically relevant fluxes.^7^

Mass spectrometry imaging (MSI) of excised tissue sections acquires a mass spectrum for each pixel of the image. The ability to perform chemical imaging in a nontargeted and label‐free way^8^ makes it a good discovery tool in the field of biology and biomedicine.^9^ The distribution of particular ions can be visualized, or a more explorative approach can be employed, leveraging clustering analysis to delineate regions of similar metabolic phenotype. In contrast to MRI, the higher spatial resolution enables direct comparison between the MS image and histological images of the same or adjacent tissue sections. MSI has been used to search for novel tumor biomarkers and to better visualize tumor margins.^10^, ^11^ The limitations of MSI include a strong dependence of the signals on sample collection and processing and the difficulty of acquiring quantitative data due to the dependence of signal intensity on the chemical and morphological environment of the location of interest.^12^, ^13^, ^14^

Combining ^13^C MRSI of hyperpolarized ^13^C‐labeled substrates with MSI offers new opportunities for investigating tumor metabolic phenotypes. Correlating the high spatial resolution MS images with the dynamic 3D MRS images acquired following injection of hyperpolarized [1‐^13^C]pyruvate can provide a better understanding of the underlying biology affecting lactate labeling kinetics while at the same time providing an opportunity to cross validate the 2 techniques.

## 2 METHODS

### 2.1 Animal experiments

Experiments were performed in compliance with a project license issued under the Animals (Scientific Procedures) Act of 1986. For validation of a rapid freezing method, kidneys of female C57BL/6 mice were excised and either freeze‐clamped using liquid nitrogen‐cooled tongs or immersed in liquid nitrogen‐cooled isopentane. For the tumor model, female C57BL/6 mice were injected subcutaneously with 5 × 10^6^ EL4 murine lymphoma cells in 200 μL fetal bovine serum and the tumors grown for 8 to 11 days.

### 2.2 Phosphorus‐31 NMR of tissue extracts

Kidney samples were homogenized in a Precellys homogenizer (Bertin Instruments, Montigny‐le‐Bretonneux, France) with 10 µL/mg 2M perchloric acid, centrifuged at 13 thousand g for 2 minutes at 4°C and the supernatant neutralized using 2M potassium hydroxide. The supernatants were lyophilized and dissolved in 550 µL deuterium oxide containing methylenediphosphonic acid at 100 nmol/g tissue. Proton decoupled phosphorus‐31 NMR spectra were acquired into 32,768 data points with a 90° pulse, 7.2 s TR, and spectral width of 57 ppm (14,006 Hz) and were the sum of 8 thousand transients.

### 2.3 Hyperpolarized [1‐^13^C]pyruvate imaging

44 mg [1‐^13^C]pyruvic acid (Cambridge Isotope Laboratories, Tewksbury, MA) were mixed with 15 mM OX063 radical (GE Healthcare, Amersham, UK) and 1.4 mM gadoterate meglumine (Dotarem, Guerbet, Villepinte, France) and polarized for ~ 1 h in a HyperSense polarizer (Oxford Instruments, Abingdon, UK) before being dissolved in 6 mL superheated buffer (40 mM Hepes, 94 mM NaOH, 30 mM NaCl, 100 mg/L ethylenediaminetetraacetic acid) at 10 bar, and 400 μL were injected immediately into the mouse tail vein.

T_2_‐weighted axial hydrogen‐1 (^1^H) ^1^H MR images (16 × 40 × 40 mm^2^, 1.25 mm thick slices) were acquired with a fast spin echo sequence. Images were reconstructed from 8 echoes with a 256 × 256 data matrix, TR 2 s, TE 48 ms. Following intravenous injection of hyperpolarized [1‐^13^C]pyruvate, ^13^C images were acquired with a single‐shot 3D volume imaging sequence.^15^ Spectral spatial pulses excited alternately the [1‐^13^C]pyruvate and [1‐^13^C]lactate resonances, with flip angles of 7° and 45°, respectively. Signal was acquired from a 40 × 40 × 20 mm^3^ volume with a matrix size of 32 × 32 × 16 data points using a stack of spiral acquisitions. The TR was 1 s so that each metabolite was imaged every 2 s over a period of 90 s. The eighth pair of acquisitions were performed without phase encoding in the third dimension and were used as a reference scan for image reconstruction.

### 2.4 Mass spectrometry imaging

Mice were snap‐frozen in liquid nitrogen‐cooled isopentane immediately following the MRI acquisition. Ten‐μm thick sections of animals injected with [1‐^13^C]pyruvate and noninjected controls were cut on a Leica cryostat (Leica Biosystems, Nussloch, Germany) and thaw‐mounted onto superfrost slides (Thermo Scientific, Bremen, Germany). The sections were air‐dried and vacuum‐packed for storage at −80°C prior to MSI.^16^ For MSI, the slides were warmed to room temperature and immersed in chloroform for 1 min 45 s. MSI was performed using a Q‐Exactive plus mass spectrometer (Thermo Scientific) with an automated desorption electrospray ionization (DESI) ion source (Prosolia Inc., Zionsville, IN). Data were acquired in negative ion mode between m/z 80 and 500. The nominal mass resolution was set to 70 thousand. The injection time was 150 ms, resulting in a scan rate of 3.8 pixel/s. A home‐built DESI sprayer^17^ was operated with a mixture of 95% methanol/5 % water at 1.5 µL/min and nebulized with nitrogen at a back pressure of 6 bar. The spatial resolution was 100 µm. Sections were then stained with hematoxylin and eosin and imaged on an Aperio scanner (Leica Biosystems, Wetzlar, Germany).

### 2.5 Data analysis

MRI data were processed using an in‐house MatLab (version R2019b, MathWorks, Natick, MA) script.^15^ The MSI .raw data files were converted into .mzML files using ProteoWizard msConvert version 3 (open source)^18^ and compiled into an .imzML file.^19^ Subsequent data processing was performed in SCiLS Lab (version 2019c, Bruker Daltonik, Bremen, Germany). Data were analyzed following RMS normalization to compensate for pixel‐to‐pixel variability and data variation between experiments.^20^ The relative distributions of [^12^C] and [1‐^13^C]lactate were visualized following hotspot removal and weak denoising.

The [1‐^13^C]lactate MS images were coregistered with the ^1^H MRI anatomical images using a custom MatLab script (version R2019b, MathWorks). The tumor in the MS image and the corresponding anatomical MR image were contoured manually to yield binary masks, which were first coregistered by affine transformation using the imregister() function. The registration was then fine‐tuned with the imregdemons() function. Nonspatial and spatial statistical techniques were used to quantify the level of association between the coregistered images. For each section, the level of association between the tumor [1‐^13^C]lactate MS and MRS images was defined by means of the Spearman rank correlation on the raw pixel intensities (estimated by means of the cor function in R) and the Cohen kappa coefficient^21^, ^22^ on standardized intensities (obtained by categorizing the raw intensities in 20 quantile based levels, estimated by means of the function cohen.kappa of the R package “psych”). One‐sided *t* tests were used to assess if the mouse mean association levels were greater than 0 (by means of the t.test R function). Linear mixed models (fitted using the lme function of R package nlme) were then used to predict the standardized MSI intensities by means of the MRI intensities, with mice and tumors as hierarchical random effects to take the within‐mouse and within‐tumor dependence into account and assuming a spherical correlation structure for the residuals.^23^, ^24^ Sensitivity analyses considering other spatial dependence structures led to similar conclusions. Significance of the relationship between MRI and MSI was assessed by means of a Wald *t* test on the MRI fixed effect parameter estimate.

Pearson and Spearman correlation coefficients were used to assess the correlation between the [1‐^13^C]lactate and [1‐^13^C]pyruvate MR images and Pearson correlation coefficients between the [^12^C] and [1‐^13^C]lactate MS images.^25^ Voxel signal intensities in the dynamic ^13^C MRSI data were summed for all those time points where the signal was greater than 10% of the maximum signal. For each slice, a region of interest was drawn around the tumor, and the corr2 MatLab function (version R2019b, MathWorks) was used to calculate the Pearson and Spearman correlation coefficients between the summed lactate and pyruvate signal intensities for that individual slice.

## 3 RESULTS

**FIGURE 1:**
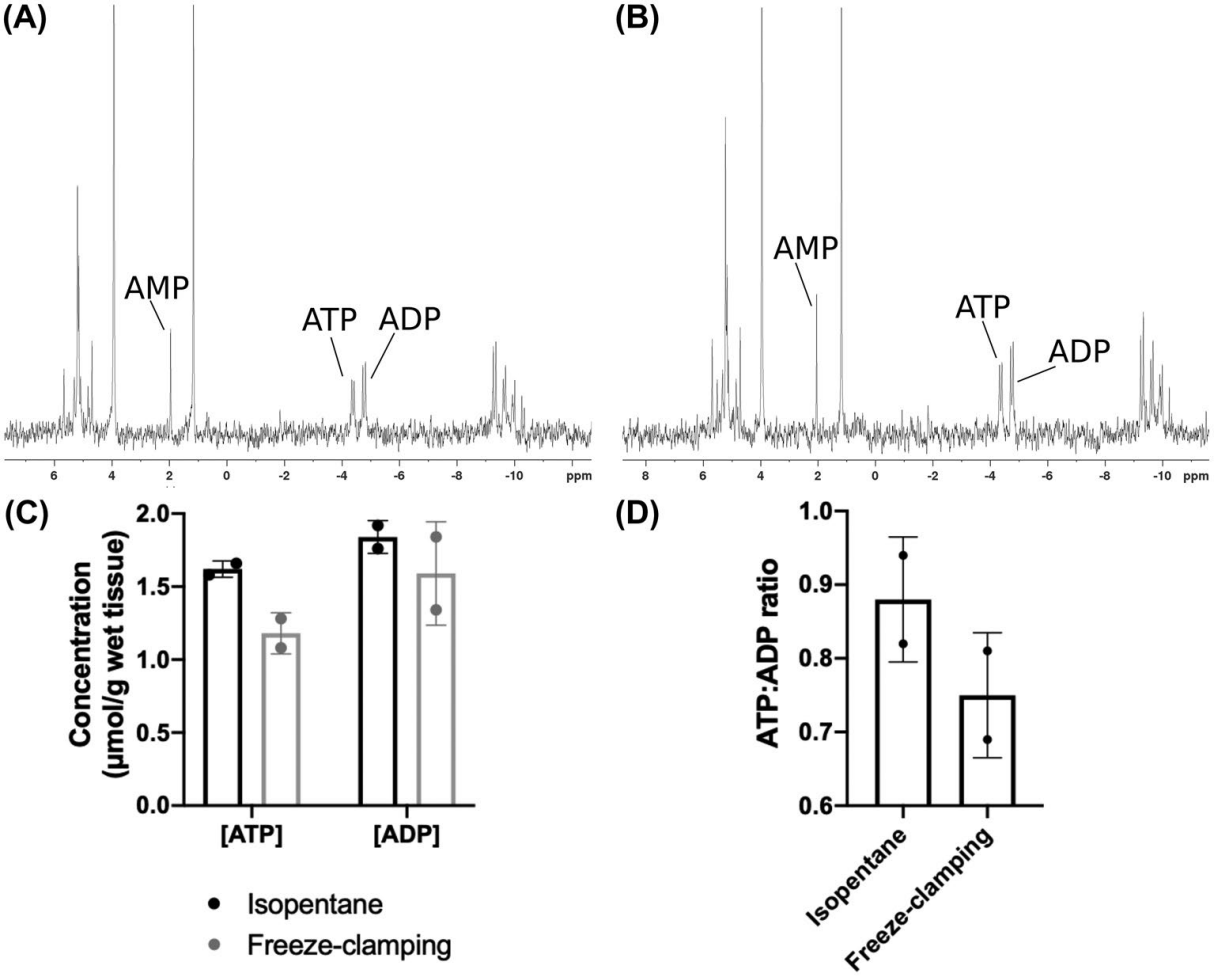
Assessment of the efficiency of freezing protocols for rapid arrest of tissue metabolism. Representative ^31^P NMR spectra of kidney extracts where paired kidneys had been freeze‐clamped using liquid nitrogen‐cooled tongs (A) or immersed in liquid nitrogen‐cooled liquid isopentane (B). The concentrations of ATP and ADP and the ATP:ADP ratio in kidneys frozen by clamping or immersion are shown in (C) and (D), respectively. ^31^P, phosphorus‐31; ADP, adenosine diphosphate; AMP, adenosine monophosphate; ATP, adenosine triphosphate

**FIGURE 2:**
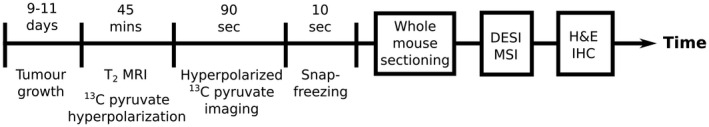
Anatomical imaging was performed while [1‐^13^C]pyruvate was being hyperpolarized. Following dissolution, the hyperpolarized [1‐^13^C]pyruvate was injected into the tail vein of the mouse and spectroscopic imaging performed. Immediately after image acquisition, the mouse was snap‐frozen, followed by whole mouse sectioning, MS, and histological imaging. Abbreviations: ^13^C, carbon‐13; DESI MSI, desorption electrospray ionization mass spectrometric imaging; H&E, hematoxylin and eosin; IHC, immunohistochemistry

A comparison of the freezing efficiency of immersion in liquid isopentane versus freeze‐clamping was performed by freezing mouse kidneys using the 2 techniques and measuring the adenosine triphosphate:adenosine diphosphate ratio in the resulting extracts using phosphorus‐31 NMR. Both techniques preserved a relatively high adenosine triphosphate:adenosine diphosphate ratio, demonstrating fast arrest of kidney metabolism (Figure 1). Immersion in precooled liquid isopentane was preferred as it preserved tissue architecture. Therefore, for subsequent experiments, mice with subcutaneous EL4 tumors were snap‐frozen by direct immersion in liquid nitrogen‐cooled isopentane (Figure 2).

**FIGURE 3:**
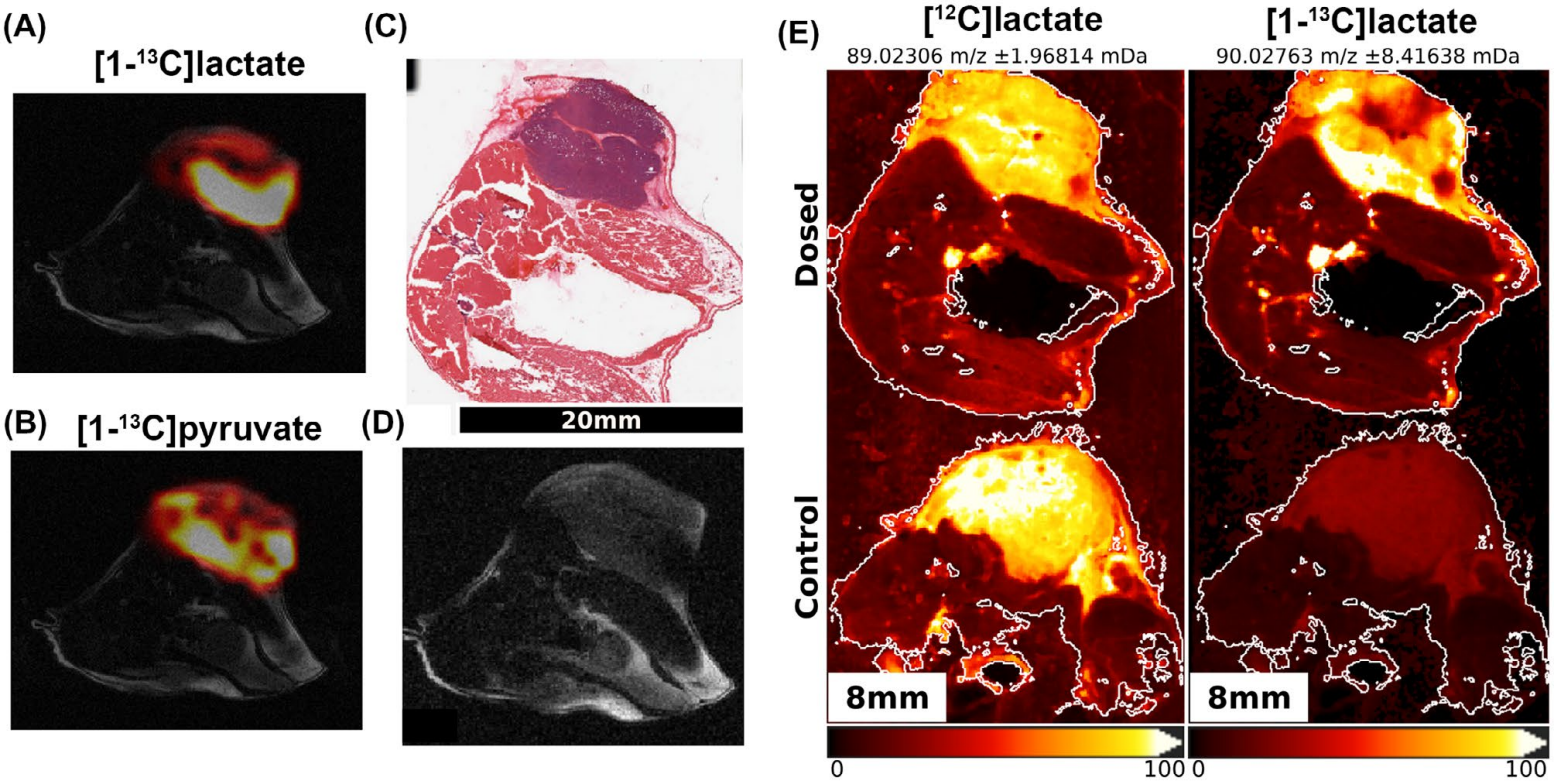
A single‐shot 3D ^13^C MRSI sequence was used to acquire [1‐^13^C]lactate (A) and [1‐^13^C]pyruvate images (B) following injection of hyperpolarized [1‐^13^C]pyruvate (images from a single slice are shown). ^13^C image intensities were normalized to the maximum intensity across the whole tumor volume, and images of the summed signal over time for each of the 16 individual slices were overlaid on the corresponding anatomical T_2_‐weighted axial ^1^H images (D). (E) Maps of [^12^C] and [1‐^13^C]lactate generated by MSI of a frozen section taken from the same slice from which the ^13^C MRS images shown in (A, B, and D) were acquired. A slice from a noninjected control mouse is also shown for comparison. The MS images were coregistered with those generated by ^13^C MRSI by manual registration in the z‐direction of the histological (C) and T_2_‐weighted MR images (D) using anatomical landmarks and the tumor outline. ^1^H, hydrogen‐1. ^12^C, carbon‐12

**FIGURE 4:**
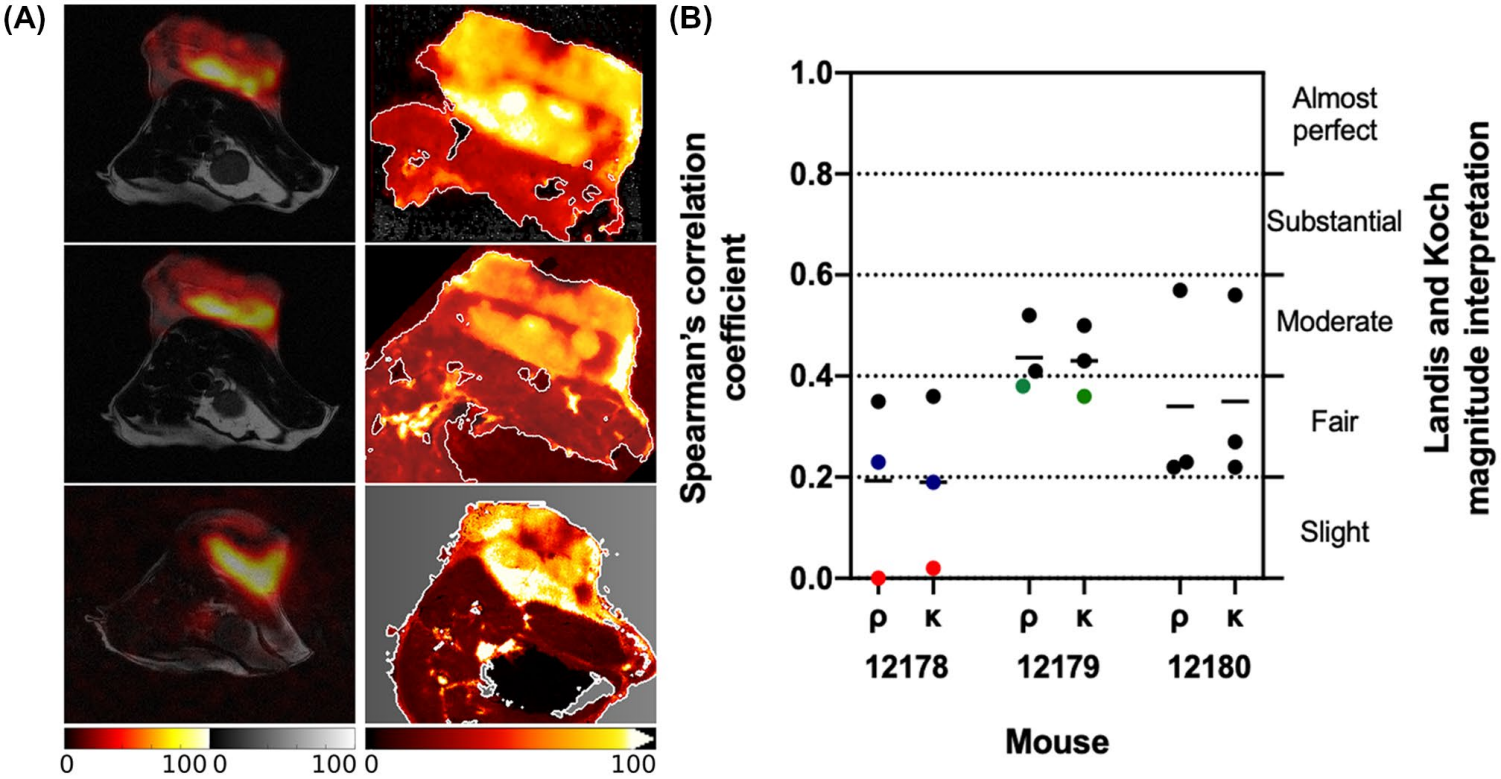
Correlation of metabolic images generated by MSI with images of hyperpolarized [1‐^13^C]lactate generated by ^13^C MRSI. A, Paired [1‐^13^C]lactate MRSI slices and corresponding MSI sections from 3 of the mice used in the study (the lowest pair of images are the same as those in Figure 3). B, Correlation of [1‐^13^C]lactate signal intensities in the tumor region in the MS and MRS images, as quantified by pixelwise Spearman rank correlation (ρ) and Cohen kappa coefficient (κ) analysis. Each point corresponds to the correlation coefficient calculated from all tumor‐containing pixels of each section/slice comparison and the line represents the mean value. The points in blue, red, and green correspond to the coefficients calculated from the top, middle, and bottom image pairs, respectively, in (A). One‐sided 1‐sample *t* test on the mean values from different mice on both Spearman and Cohen kappa coefficients showed a statistically significant correlation (*P* = .02)

Following injection of hyperpolarized [1‐^13^C]pyruvate into an EL4 tumor‐bearing mouse, 16 axial images of [1‐^13^C]lactate and [1‐^13^C]pyruvate were acquired using a single‐shot, 3D volume imaging sequence.^15^ The subcutaneous tumors showed pyruvate uptake and conversion to lactate, with consistently a region of high ^13^C lactate signal intensity at the tumor base (Figures 3 and 4) (Supporting Information Figure S1). [1‐^13^C]lactate and [^12^C]lactate were detected in excised tumor sections by DESI MSI and metabolic maps generated from their peak intensities (Figure 3 and Supporting Information Figure S1). Animals not injected with [1‐^13^C]pyruvate served as controls for the natural isotopic abundance of [^13^C]lactate and to confirm assignment of the [1‐^13^C]lactate peak. Similarly to the MR images, the [1‐^13^C]lactate distribution measured by MSI showed higher signal intensity at the base of the tumors (Figures 3 and 4). We were unable to detect [1‐^13^C]pyruvate or unlabeled pyruvate. In previous studies in this tumor model, we were also unable to detect ^13^C‐labeled or unlabeled pyruvate using ^1^H and ^13^C NMR measurements on tumor extracts.^7^

The ^13^C MR images of hyperpolarized [1‐^13^C]lactate were compared with the [1‐^13^C]lactate MS images of the corresponding excised tissue sections. For each mouse, at least 3 sections were used. The ^1^H MRI slice corresponding to each MSI section was manually selected using anatomical landmarks within the tumor (Figure 3). The selected slices from the T_2_‐weighted ^1^H images, which served as reference images for the ^13^C MRS images, were then coregistered with the MS images. The positive correlation between the ^13^C lactate MS and MR images, as measured by means of Spearman rank correlation and Cohen weighted kappa estimates, was statistically significant (Figure 4). Based on the Landis and Koch interpretation of Cohen weighted kappa coefficients,^26^ the association in this case is “fair” on average. This is expected given that the data were generated by different modalities with different spatial resolutions, and more importantly, the comparison was made between intact tissue in vivo and frozen tissue sections ex vivo. To further validate the significance of the association level between the images generated by the 2 modalities, a linear mixed model—a more powerful parametric analysis able to model the within‐mouse and within‐tumor, and spatial dependence of the MR and MS pixel intensities—was fitted. This showed a highly significant relationship between the MR and MS pixel intensities (*P* value of 1.75*10^−9^) but a marginal R‐squared—fraction of variance of MS intensities explained by the MR ones—of 0.11.

**FIGURE 5:**
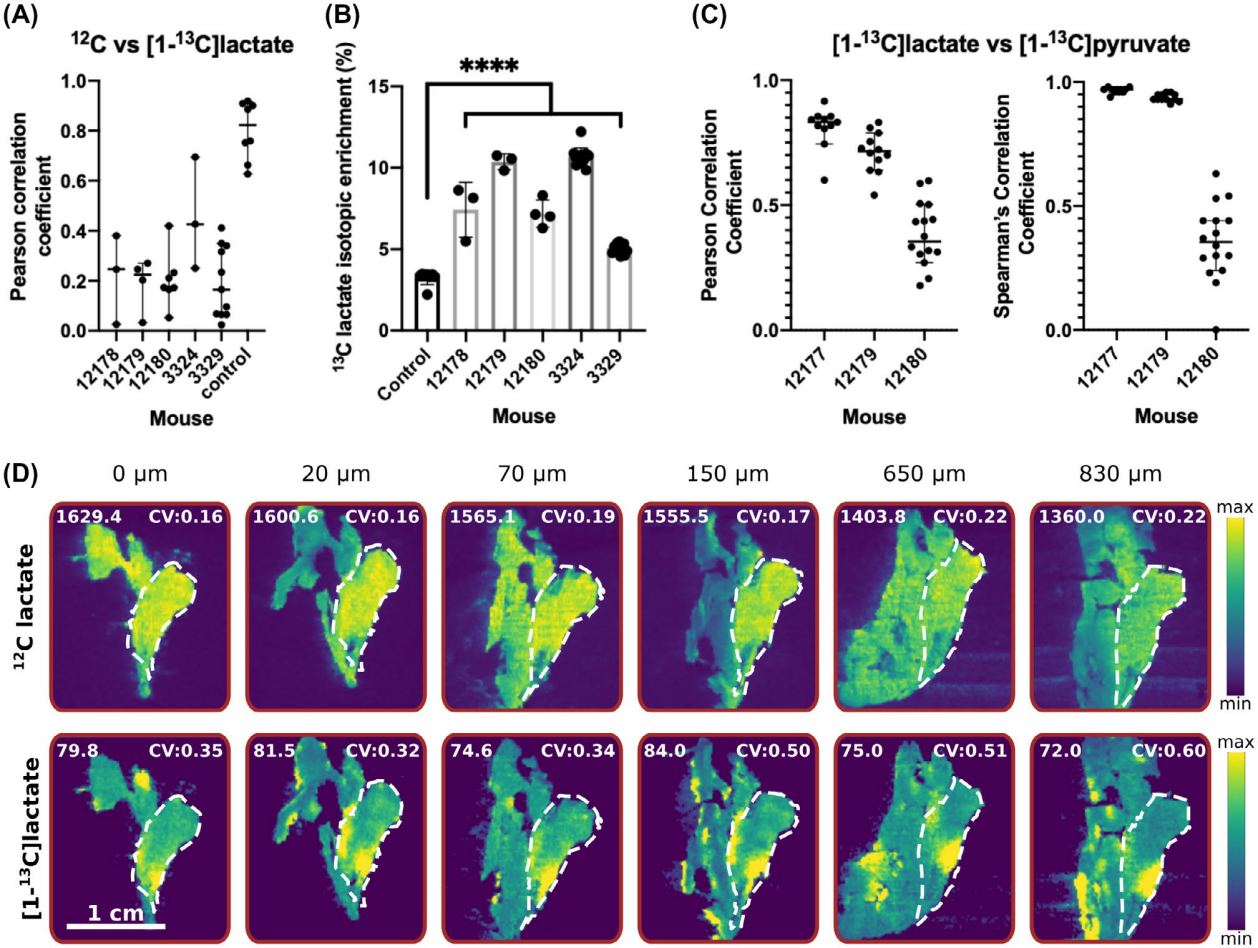
A, Pearson correlation coefficients between endogenous unlabeled lactate ([^12^C]lactate) and [1‐^13^C]lactate across all tumor‐containing pixels from all scanned MSI sections for 5 mice injected with hyperpolarized [1‐^13^C]pyruvate and a control noninjected mouse. The line and bars represent the median correlation coefficient and 95% CI, respectively. B, The percentage [^13^C]lactate isotopic enrichment for control, noninjected animals, and animals injected with hyperpolarized [1‐^13^C]pyruvate. Each point represents quantitation of a different section, and the error bars represent the SD on the mean. Dunnet multiple comparisons test was used to compare the isotopic enrichment of experimental animals with the control animal. ****P < .0001. C, Pearson and Spearman correlation coefficients between [1‐^13^C]lactate and [1‐^13^C]pyruvate signal intensities in tumor‐containing voxels in the ^13^C MRS images. Each point corresponds to the correlation coefficient for an individual MRI slice. The line and bars represent the median correlation coefficient and 95% CI, respectively. D, [^12^C] and [1‐^13^C]lactate signal intensities in MS images of 6 sections from a 1 mm slice. The intensities are expressed relative to the maximum signal intensity of the individual species. Tumor regions are indicated by a dashed white line. The mean signal intensity of each lactate species across the tumor is indicated for each section as well as the CV. CI, confidence interval; CV, coefficient of variance

The [^12^C]lactate distribution, as determined by MSI, was consistently different from the [1‐^13^C]lactate distribution determined by ^13^C MRSI (Figure 3), indicating that lactate labeling does not simply reflect the endogenous lactate pool size. This was confirmed by comparing the [^12^C] and [1‐^13^C]lactate distributions in the MS images, where the low Pearson correlation coefficients confirmed that lactate labeling was not strongly dependent on the endogenous lactate concentration (Figure 5A). A positive control was provided by a noninjected animal, which showed a good correlation between the natural abundance ^13^C signal and unlabeled lactate ([^12^C]lactate). The measured lactate isotopic enrichment in control tumors matched the expected theoretical enrichment, and the additional enrichment following injection of hyperpolarized [1‐^13^C]pyruvate matches what we have measured previously in this tumor model^7^ (Figure 5B). Further confirmation came from comparing MS images of several axial sections corresponding to a single MRI slice, where there was no correlation between the distribution of [^12^C]lactate and [1‐^13^C]lactate, with the former being more homogeneously distributed than the latter (Figures 5D and S3). Conversely, the ^13^C MRS images of hyperpolarized [1‐^13^C]pyruvate and [1‐^13^C]lactate showed a significantly stronger positive correlation (Figure 5C), suggesting that ^13^C lactate labeling is limited principally by the availability of labeled pyruvate in this tumor model. Lactate labeling could also be affected by the levels of the coenzyme, nicotinamide adenine dinucleotide (NAD+) and its reduced form (NADH); however, matrix‐assisted laser desorption mass spectrometric imaging showed that nicotinamide adenine dinucleotide (NAD+) and its reduced form (NADH) were homogeneously distributed in these tumors (Supporting Information Figure S2), consistent with the relatively uniform distribution of unlabeled lactate (Figures 3 and 5).

## 4 DISCUSSION

Dynamic ^13^C MRSI was used to measure the rate of interconversion of pyruvate and lactate in vivo, with a time resolution of 2 s, a nominal spatial resolution of 1.25 × 1.25 × 1.25 mm^3^, and an effective resolution of 1.25 × 1.25 × 4.5 mm^3^ when taking into account the z‐direction point spread function.^15^ The EL4 tumors showed rapid [1‐^13^C]pyruvate uptake and very fast conversion to [1‐^13^C]lactate, as has been observed previously in this tumor model.^27^, ^28^ Sixteen axial ^13^C MRSI slices were acquired from each of 3 EL4 tumors and spatial maps of [1‐^13^C]lactate and [1‐^13^C]pyruvate were overlaid on the anatomical T_2_‐weighted ^1^H MR images. The [1‐^13^C]lactate signal was largely localized to the tumor and showed a heterogeneous distribution. The mice were snap‐frozen immediately after the MRI acquisition using a freezing technique that rapidly arrested metabolism. The [1‐^13^C]lactate MS images were thus expected to show a good correlation with the summed [1‐^13^C]lactate images produced by ^13^C MRSI; indeed, there was agreement between the 2 (Figure 4).

The significant association between the ^13^C lactate maps generated by DESI and ^13^C MRSI suggest that the ionization efficiency was consistent across the whole tumor section. The observed positive correlation between the datasets validates annotation of the metabolites in the MSI data in which isobaric species with the same chemical formula cannot be distinguished from one another. The assignments for the [1‐^13^C]lactate peak were further validated using negative control tissues, which contained only naturally abundant [^13^C]lactate.

Coregistration of the MRS and MS images was assisted by taking whole mouse axial sections for MSI. However, changes in morphology due to rapid freezing, the difficulty of taking intact sections through the whole frozen mouse body, and the differences in the thickness of the MSI (10 μm) and MRSI (1.25 mm) slices meant that the excised sections did not match perfectly the MR images. Anatomical landmarks in the tumor, which appeared in both the ^1^H images and in the hematoxylin and eosin‐stained sections, were used to facilitate manual assignment of MS images to their corresponding MRI slice. The coregistration MatLab (version R2019b, MathWorks) routines were successful in registering the tumor masks, defined by drawing a region of interest around the tumor, by applying rigid and nonrigid transformations. However, in most cases, the tumor outline was distorted, thus limiting the accuracy of the registration. Therefore, the magnitude of the correlation between MS and MR images is an underestimate of the agreement between the 2 imaging modalities. The statistical significance for the correlation between the 2 modalities was confirmed using spatial and nonspatial statistical tools, enabling cross‐validation of the hyperpolarized ^13^C MRI and MSI techniques.

The exchange of ^13^C label between pyruvate and lactate in a tumor is dependent on [1‐^13^C]pyruvate delivery, cell uptake via the monocarboxylate transporters 1 and 4 (MCT1 and MCT4), the concentrations of lactate dehydrogenase and its coenzymes, and the size of the endogenous tumor lactate pool.^30^ The relative importance of these factors will likely vary according to tumor type. Previous studies on EL4 cells showed that the activities of the MCTs and lactate dehydrogenase are nearly equally important in determining the exchange velocity.^29^ Using MSI, we have shown that in EL4 tumors, the distribution of endogenous tumor lactate and NAD(H) were poorly correlated with lactate labeling but that there was a stronger correlation between the [1‐^13^C]pyruvate and [1‐^13^C]lactate signals in the ^13^C MRS images, suggesting that delivery of labeled pyruvate has the greatest effect on the exchange velocity under these conditions. This does not mean that the activities of the MCTs and lactate dehydrogenase are unimportant; however, it suggests that under these conditions and in this tumor model, pyruvate delivery is more important. A recent clinical study in prostate cancer patients showed positive correlation between MCT1 expression and lactate labeling,^3^ and we have shown previously in the EL4 tumor model that the MCT inhibitor, α‐Cyano‐4‐hydroxycinnamic acid (injected at 150 mg kg^1^), produced a 40% decrease in label exchange.^30^

## 5 CONCLUSION

Hyperpolarized [1‐^13^C]pyruvate imaging and MSI generated positively correlated tumor images of [1‐^13^C]lactate following injection of hyperpolarized [1‐^13^C]pyruvate, thus cross‐validating the 2 techniques. The [1‐^13^C]lactate maps in the tumor were poorly correlated with the endogenous tumor lactate levels but highly correlated with maps of [1‐^13^C]pyruvate, suggesting that in this tumor pyruvate delivery has the greatest effect on the rate of lactate labeling.

## CONFLICT OF INTEREST

K.M.B. holds patents and has research agreements with GE Healthcare that are relevant to this paper.

## Supporting information


**FIGURE S1** A single shot three‐dimensional ^13^C MRSI sequence was used to acquire interleaved [1‐^13^C]pyruvate (A) and [1‐^13^C]lactate images (B). Each image is from a nominal 1.25 mm thick axial slice. ^13^C image intensities were normalized to the maximum intensity of the respective metabolite across the whole volume. These images and the time course of metabolite labeling (C) are from a representative tumor. In (C) absolute signal intensities are shown after summing signal in the tumor region from all the slices. The signal intensity for lactate is much higher than that for pyruvate reflecting the larger flip angle pulse used to excite the lactate resonance. (D) [^12^C] and [1‐^13^C]lactate were detected using negative ion mode DESI MSI. The average spectrum from a single section is shown, with the endogenous lactate peak and a smaller ^13^C lactate peak appearing at 89 and 90 m/z ratios, respectively
**FIGURE S2** Representative images of NAD+ (664.1 ppm) and NADH [M+H+] adduct (666.1 ppm)^2^ are shown for three different animals. The middle two columns are from the same animal. False color images represent the relative intensity of the indicated ion. The bottom panel shows an overlay of the relative intensities of NAD+ and NADH, where the colocalization of the molecules leads to a yellow color. The tumor region is indicated by the dashed white line
**FIGURE S3** Representative MS images of [^12^C] and [1‐^13^C]lactate signal intensities in six sections from a 1 mm slice. The intensities are expressed relative to the maximum signal intensity of the individual species. Tumor regions are indicated by a dashed white line. The mean signal intensity of each metabolite across the tumor is indicated for each section as well as the Coefficient of Variance (CV)
