# Supplementary material for: Comparison of 13C MRI of hyperpolarized [1‐13C]pyruvate and lactate with the corresponding mass spectrometry images in a murine lymphoma model
Source: Magn Reson Med. 2021 Jan 9;85(6):3027–35. doi: 10.1002/mrm.28652 (PMC7986146; doi:10.1002/mrm.28652)
Supplement: Supplementary file 1 — FIGURE S1 A single shot three‐dimensional 13C MRSI sequence was used to acquire interleaved [1‐13C]pyruvate (A) and [1‐13C]lactate images (B). Each image is from a nominal 1.25 mm thick axial slice. 13C image intensities were normalized to the maximum intensity of the respective metabolite across the whole volume. These images and the time course of metabolite labeling (C) are from a representative tumor. In (C) absolute signal intensities are shown after summing signal in the tumor region from all the slices. The signal intensity for lactate is much higher than that for pyruvate reflecting the larger flip angle pulse used to excite the lactate resonance. (D) [12C] and [1‐13C]lactate were detected using negative ion mode DESI MSI. The average spectrum from a single section is shown, with the endogenous lactate peak and a smaller 13C lactate peak appearing at 89 and 90 m/z ratios, respectively FIGURE S2 Representative images of NAD+ (664.1 ppm) and NADH [M+H+] adduct (666.1 ppm) 2 are shown for three different animals. The middle two columns are from the same animal. False color images represent the relative intensity of the indicated ion. The bottom panel shows an overlay of the relative intensities of NAD+ and NADH, where the colocalization of the molecules leads to a yellow color. The tumor region is indicated by the dashed white line FIGURE S3 Representative MS images of [12C] and [1‐13C]lactate signal intensities in six sections from a 1 mm slice. The intensities are expressed relative to the maximum signal intensity of the individual species. Tumor regions are indicated by a dashed white line. The mean signal intensity of each metabolite across the tumor is indicated for each section as well as the Coefficient of Variance (CV) [file MRM-85-3027-s001.docx]

Comparison of ^13^C magnetic resonance images of hyperpolarized
[1-^13^C]pyruvate and lactate with the corresponding mass spectrometry images in a murine lymphoma model

Maria Fala^1^, Vencel Somai^1,2^, Andreas Dannhorn^3^, Gregory Hamm^3^, Katherine Gibson^3^, Dominique-Laurent Couturier^1^, Richard Hesketh^1^, Alan J. Wright^1^, CRUK Rosetta Consortium^4^, Zoltan Takats^5^, Josephine Bunch^6^, Simon T Barry^7^, Richard J. A Goodwin^3,9^, Kevin M. Brindle^1,8*^

^1^Cancer Research UK Cambridge Institute, University of Cambridge, Li Ka Shing Centre, Robinson Way, Cambridge, UK

^2^Department of Radiology, University of Cambridge, School of Clinical Medicine Box 218, Cambridge Biomedical Campus, UK

^3^Imaging and Data Analytics, Clinical Pharmacology and Safety Sciences R&D, AstraZeneca, Cambridge CB4 0WG, UK

^4^CRUK Grand Challenge Rosetta Consortium

^5^Department of Digestion, Metabolism and Reproduction, Sir Alexander Fleming Building, Imperial College London, London SW7 2AZ, UK

^6^National Centre of Excellence in Mass Spectrometry Imaging (NiCE-MSI), National Physical Laboratory, Teddington TW11 0LW, UK

^7^Bioscience, Discovery, Oncology R&D, AstraZeneca, Cambridge, United Kingdom

^8^Department of Biochemistry, University of Cambridge, Tennis Court Road, Cambridge, United Kingdom

^9^Institute of Infection, Immunity and Inflammation, College of Medical, Veterinary and Life Sciences, University of Glasgow, Glasgow G12 8QQ, UK

**Supporting Information Figures**

**Supporting Information Figure S1**


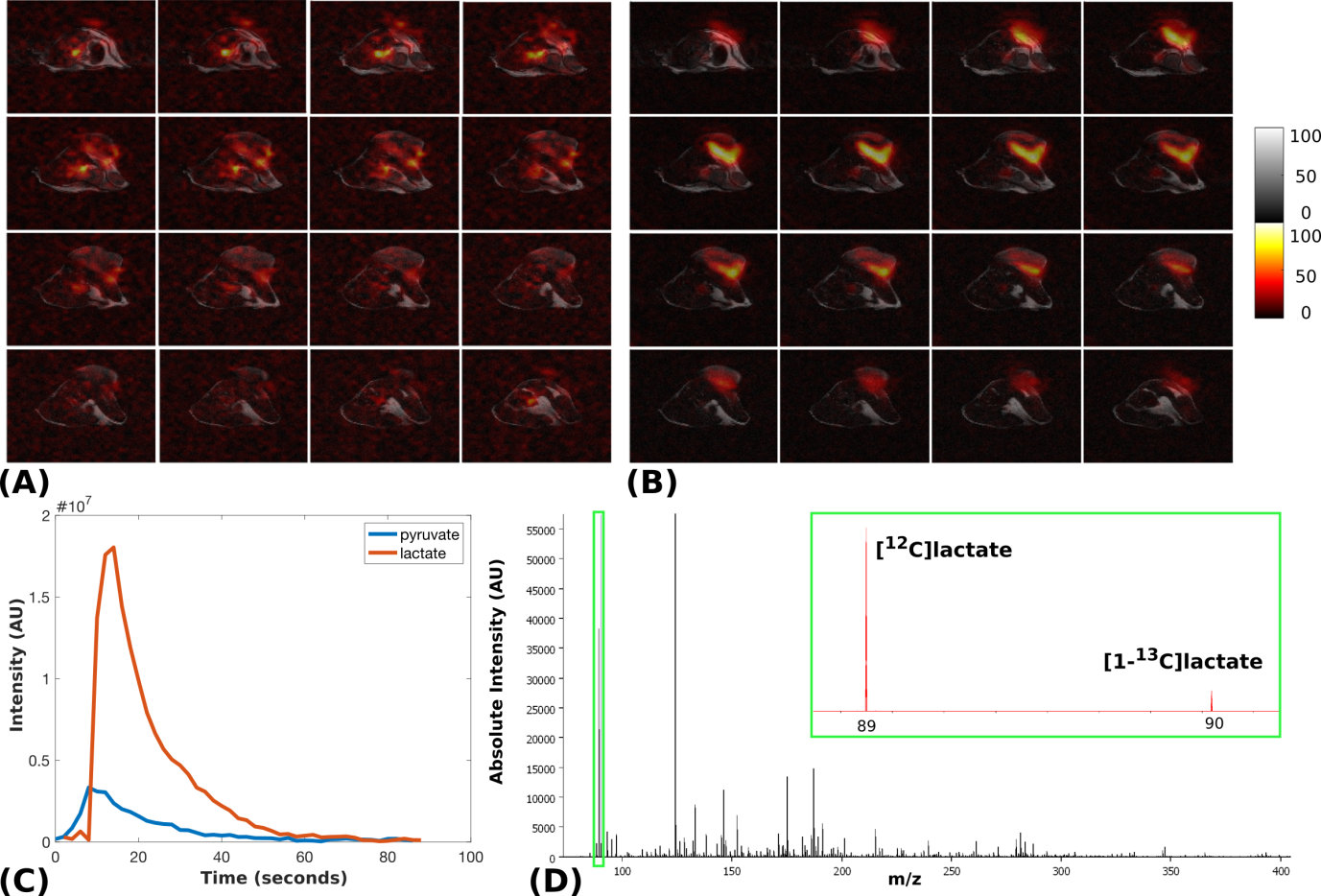


A single shot three-dimensional ^13^C MRSI sequence was used to acquire interleaved [1-^13^C]pyruvate (A) and [1-^13^C]lactate images (B). Each image is from a nominal 1.25 mm thick axial slice. ^13^C image intensities were normalized to the maximum intensity of the respective metabolite across the whole volume. These images and the time course of metabolite labeling (C) are from a representative tumor. In (C) absolute signal intensities are shown after summing signal in the tumor region from all the slices. The signal intensity for lactate is much higher than that for pyruvate reflecting the larger flip angle pulse used to excite the lactate resonance. (D) [^12^C] and [1-^13^C]lactate were detected using negative ion mode DESI MSI. The average spectrum from a single section is shown, with the endogenous lactate peak and a smaller ^13^C lactate peak appearing at 89 and 90 m/z ratios, respectively.

**Supporting Information Figure S2**


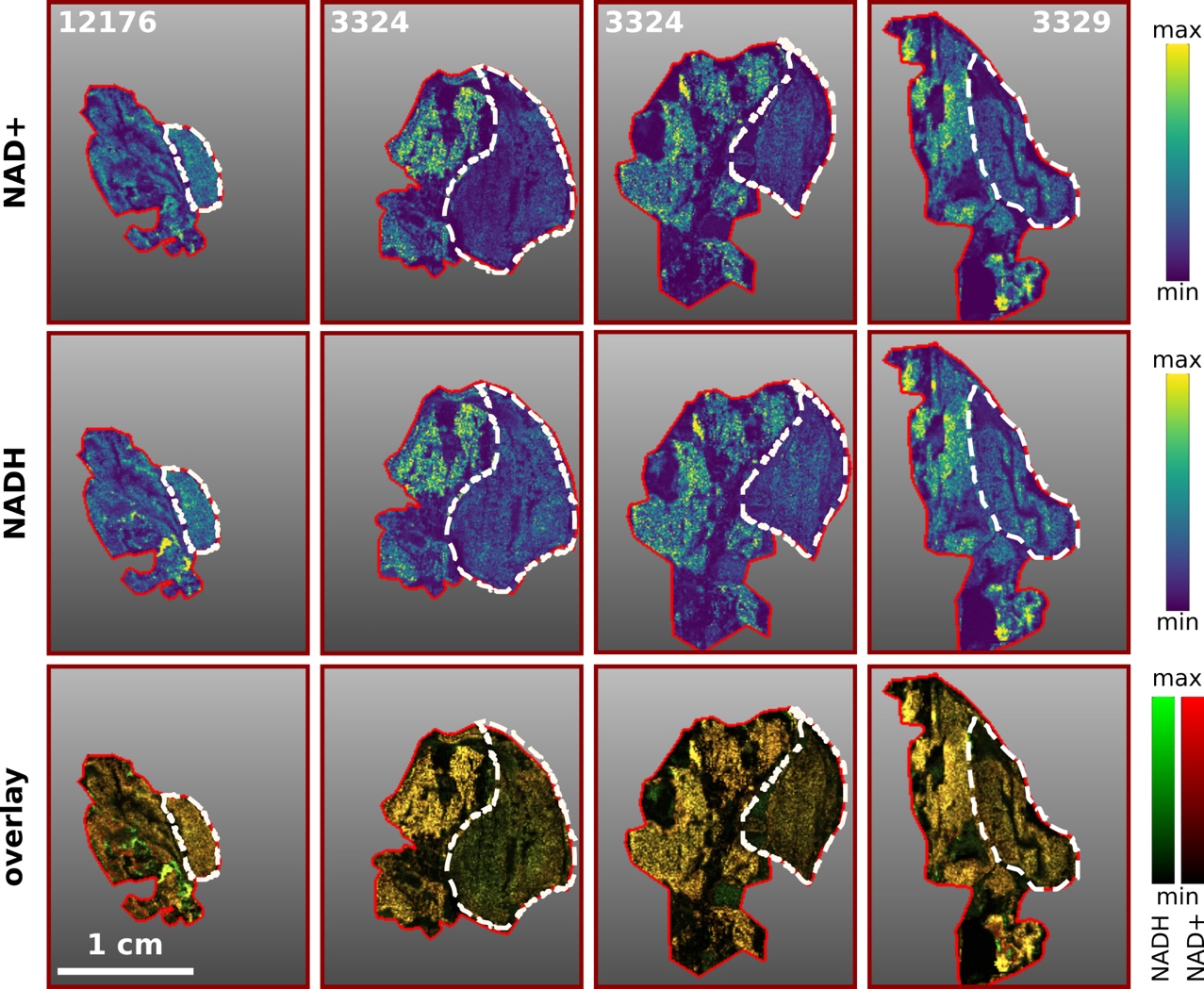


Representative images of NAD+ (664.1 ppm) and NADH [M+H+] adduct (666.1 ppm) (2) are shown for three different animals. The middle two columns are from the same animal. False color images represent the relative intensity of the indicated ion. The bottom panel shows an overlay of the relative intensities of NAD+ and NADH, where the colocalization of the molecules leads to a yellow color. The tumor region is indicated by the dashed white line.

**Supporting Information Figure S3**


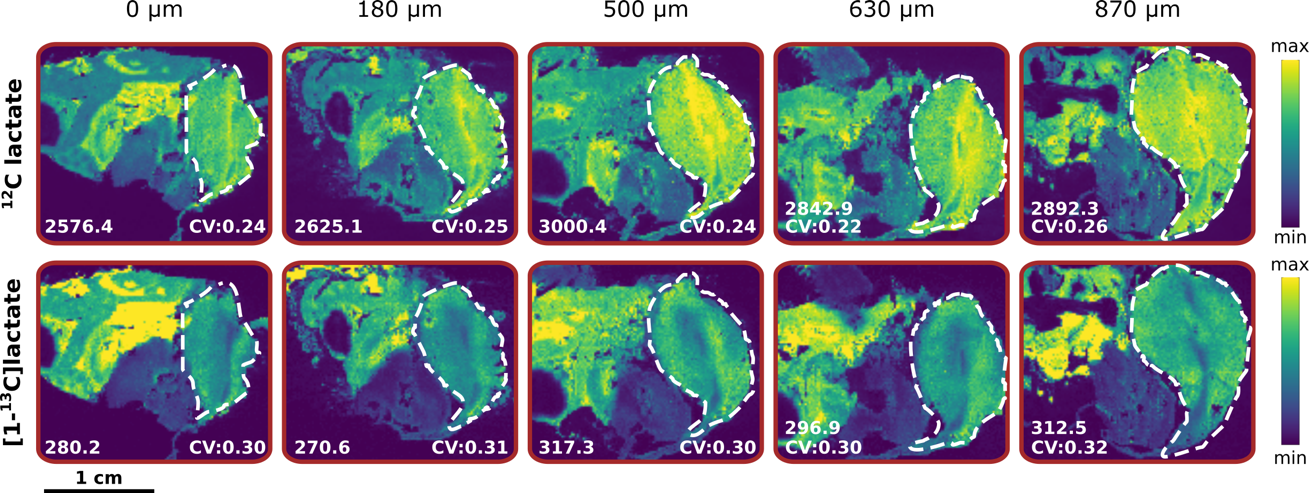


Representative MS images of [^12^C] and [1-^13^C]lactate signal intensities in six sections from a 1 mm slice. The intensities are expressed relative to the maximum signal intensity of the individual species. Tumor regions are indicated by a dashed white line. The mean signal intensity of each metabolite across the tumor is indicated for each section as well as the Coefficient of Variance (CV).

Matrix-assisted laser desorption/ionization (MALDI) imaging of NAD+ and NADH was performed on 10 μm thick sections cut from frozen tumors and mounted on indium tin oxide-coated slides. Analysis was performed using a RapifleX Tissuetyper instrument (Bruker Daltonik, Bremen, Germany). An automated spray system (TM-Sprayer, HTX technologies, Chapel Hill, NC, USA) was used to deposit the matrix (2,5-Dihydroxybenzoic acid prepared in 50:50:0.1 acetonitrile:water:trifluoroacetic acid) using a protocol reported previously (1). Imaging was performed with a spatial resolution of 100 µm and data were collected in the mass range between m/z 200 and 1000 in positive ion mode. A total of 750 laser shots were summed per pixel to give the final spectra. The laser was operated at a repetition rate of 10 kHz. The raw data were uploaded and processed in FlexImaging or SCiLS lab (Version 2020b) software packages (Bruker Daltonik, Bremen, Germany).

**References**

1. Swales JG, Tucker JW, Strittmatter N, Nilsson A, Cobice D, Clench MR, Mackay CL, Andren PE, Takats Z, Webborn PJ, Goodwin RJ. Mass spectrometry imaging of cassette-dosed drugs for higher throughput pharmacokinetic and biodistribution analysis. Anal Chem 2014;86(16):8473-8480.

2. Yaku K, Okabe K, Nakagawa T. Simultaneous measurement of NAD metabolome in aged mice tissue using liquid chromatography tandem-mass spectrometry. Biomedical chromatography : BMC 2018;32(6):e4205.
